# Long term stability of paraoxonase-1 and high-density lipoprotein in human serum

**DOI:** 10.1186/1476-511X-11-53

**Published:** 2012-05-14

**Authors:** Piet K Beekhof, Maryana Gorshunska, Eugène HJM Jansen

**Affiliations:** 1 Laboratory for Health Protection Research, National Institute of Public Health and the Environment, PO Box 1, Bilthoven, BA, 3720, The Netherlands; 2 Department of Endocrinology, Kharkiv Postgraduate Medical Academy, Korchagintsiv Str., 58, Kharkiv, 61176, Ukraine

**Keywords:** Paraoxonase, High-density lipoprotein, Serum, Stability, Storage

## Abstract

**Background:**

Paraoxonase-1 (PON1) is an enzyme with numerous functions and receives an increasing interest in clinical and epidemiological studies. Sometimes samples are stored for longer periods at a certain temperature. Therefore the stability of PON1 activity must be checked and retained upon storage for longer periods.

**Results:**

In this study the stability of PON1 activity has been tested in human serum samples during storage up to 12 months at 3 commonly used temperatures, -20°C, -70°C and −196°C. It was found that the stability of the PON1 activity is constant during 12 months of storage at −70°C and −196°C. Storage at −20°C resulted in a small but statistically significant decrease after 6 months to about 94% of its original value. Nonetheless, the rank order between the samples at T = 0 and 12 months remained the same. The same temperature dependence was found for the associated high-density lipoprotein.

**Conclusions:**

It can be concluded that −70°C is the right temperature for storage to maintain the PON1 activity for at least one year. Storage at a lower temperature in liquid nitrogen (−196°C) is not necessary.

## Background

In medical and epidemiological research the stability and activity of biomarkers is very important especially when they are stored for a long period of time at a certain temperature. Also standardization of processes such as blood withdrawal, centrifugation and time until storage at low temperatures is necessary. Other important parameters include the time delay until storage, the storage temperature and the storage time until analysis all contribute to the process of gathering a reliable set of data for statistical analysis.

Paraoxonase-1 (PON1) has obtained during the last years much interest in clinical and epidemiological research focusing on its protective role in vascular disease [1,2] diabetes [3-5] and end-stage renal disease [6]. PON1 is a high-density lipoprotein (HDL)-associated enzyme exhibiting anti-atherogenic properties and protects low-density lipoprotein (LDL) against oxidation in the prevention of atherosclerosis. Besides these protecting properties, PON1 exhibits a range of important activities, including drug metabolism, detoxification of organophosphates such as nerve agents and also plays a protective role as homocystein thiolactonase activity against oxidative damage of lipoproteins such as homocysteinylation [3,6] and interference with lipid metabolism [7-10].

In the present study the stability of PON1 activity and HDL-C (HDL-cholesterol) concentration in serum samples have been tested after a storage time up to 12 months at 3 different commonly used temperatures.

## Results

The initial concentrations of PON1 at t = 0 have been determined within 4 hours after centrifugation. Mean ± SD for PON1 for the 16 volunteers was 67.7 ± 16.3 U/L with a range of 43.1-109.7 U/L with a median value of 67.4 U/L.

**Figure 1:**
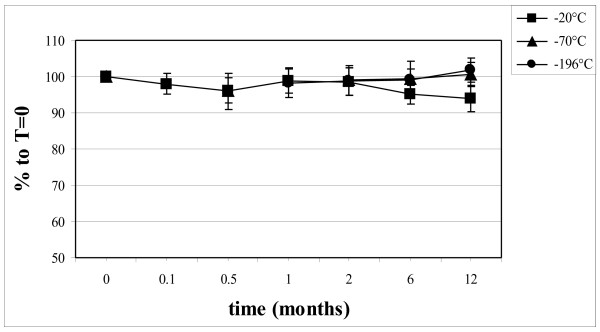
Long-time stability of PON1 activity upon storage at different temperatures, corrected for the levels of the control sample.

The time and temperature dependence of PON1 activity which was corrected with the values of the quality control sample is shown in Figure 1.

Upon storage at −20°C, there was a statistically significant decrease in PON1 activity after 6 and 12 months to 96% and 94% compared with the activity of PON1 in the samples stored at −70°C or −196°C (p < 0.01 and p < 0.001, respectively). The differences between values at −70°C and at −196°C were not statistically significant. The rank order of the PON1 activity in the serum samples after 12 months of storage at −20°C, however remained unchanged with a good correlation (R^2^) of 0.954 with the data at T = 0. Storage at −70°C or −196°C showed no decrease in the mean value of PON1 activity at all time points. The correlation coefficients with the data at T = 0 were 0.973 and 0.969 for the samples stored at −70°C and −196°C, respectively.

**Figure 2:**
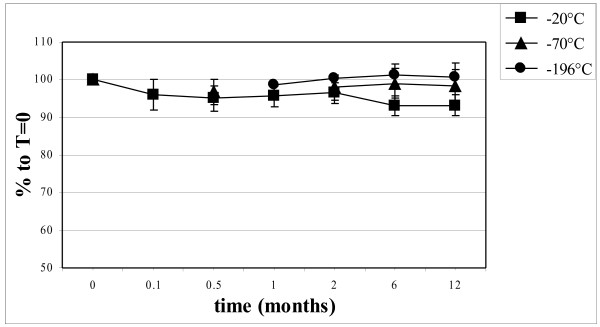
Long-time stability of HDL-cholesterol upon storage at different temperatures, corrected for the levels of the control sample.

To investigate the influence of storage on the binding of PON1 to high-density lipoprotein (HDL), the parameter HDL-cholesterol (HDL-C) was also measured. Figure 2 shows the stability of the HDL-C concentration corrected for the control sample. A similar pattern was observed as for the PON1 activity. Storage at −70°C and −196°C did not affect the HDL-C concentration, but storage at −20°C decreased the HDL-C concentration to 92% of the original value after 6 and 12 months. At these time points there was a statistically significant difference between the mean values of the samples stored at −20°C and at −196°C (p < 0.001). No statistical differences were observed between values at −70°C and at −196°C.

## Discussion

This study shows that the stability of PON1 activity is good upon long time storage up to 12 months. At time points 1, 9 and 12 months there seems to be a small decrease in the PON1 activity relative to T = 0 but after correction for the control serum data, the PON1 activity of all time points at storage temperatures of −70°C and −196°C did not deviate from the value at T = 0. Only at time points of 9 and 12 months, the activity of the samples stored at−20°C showed a statistically significant decrease in activity to 94% compared with the activities of the samples stored at −70°C and −196°C. Because the rank order compared with samples at T = 0 was very good, the PON1 activity data of samples stored at −20°C for 12 months still can be used in epidemiological research for statistical analysis.

Similar effects were observed with HDL-C after correction for the control serum data, where the HDL-C concentration after 6 and 12 months stored at −20°C showed a decrease in activity to about 92% compared with the activities of the samples stored at −70°C and −196°C. Whether this decrease in both PON1 activity and HDL-C concentration are correlated can not be determined with the present data.

The present study confirms the findings of Huen et al. [11] who also found a good stability of the PON1 activity up to 2 years at −80°C. In addition, two other temperatures (−20°C and −196°C) have been tested in this study.

## Conclusions

In conclusion, the PON1 activity in serum samples stored at −70°C or −196°C are perfectly stable during one year of storage. Upon storage at −20°C a small decrease to 96% and 94% has been observed after 6 months of storage. The same temperature dependence was observed for HDL-C. Because there were no differences found in stability of PON1 stored at −70°C and −196°C, complicated and expensive storage devices such as a container with liquid nitrogen for a storage temperature of −196°C are not necessary to maintain the activity of PON1.

## Methods

For the stability study, serum samples of 16 healthy human volunteers, 8 men and 8 women (blood donors) were used. The mean age was 43.8 years and the health status was checked with the procedures used in the Blood Bank. Samples were obtained from the Central Blood Laboratory of the Red Cross in Amsterdam with written permission of the volunteers. After blood withdrawal, serum samples were prepared within two hours, divided in aliquots and stored at different temperatures.

The initial activity of PON1 and the HDL-C concentration at t = 0 were determined within 4 hours after centrifugation. For long-term stability samples have been stored for 12 months at −20°C, -70°C or −196°C. Samples stored at −20°C were kept in a freeze room with a daily temperature check. Measurements of PON1 activity and HDL-C concentration were performed at day 4 and 14 and after 1, 2, 6 and 12 months. Samples stored at −70°C were kept in a refrigerator equipped with temperature recorder and sound alarm. Measurements of PON1 activity and HDL-C concentration were performed at day 14 and after 2, 6 and 12 months. Samples stored at −196°C were kept in a container with liquid nitrogen equipped with an automatic filling device and sound alarm. Measurements of PON1 activity and HDL-C concentration were performed after 1, 2, 6 and 12 months.

At all time points the statistical difference (95% confidence interval) of the values from the initial value at t = 0 were determined with a t-test for two-samples assuming equal variances.

**Figure 3:**
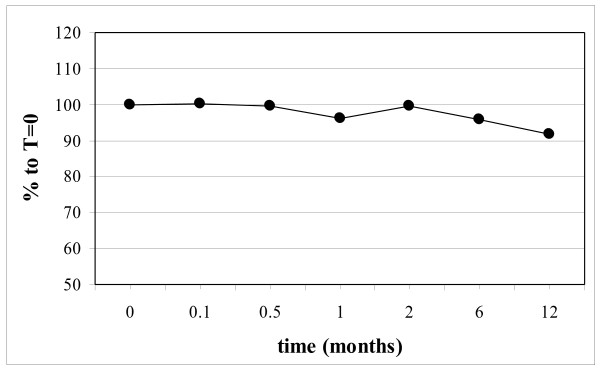
Levels of PON1 activity (single measurements) in the control sample during the course of the experiment.

PON1 activity was determined in serum on an auto analyzer (Hitachi 912, Roche Diagnostics) as described by Himbergen et al. [12]. Shortly, paraoxon was used as substrate for PON-1 in the presence of 2.3 M NaCl and 2.3 mM CaCl_2_. in Tris/HCl buffer at pH 8.5. The formation of p-nitrophenol was followed kinetically at 450 nm with 546 nm as reference wavelength. Precinorm-U has been used as a control serum. HDL-C concentrations in serum have been determined on an auto analyzer (Hitachi 912, Roche Diagnostics) with dedicated kits. The values of the control serum for PON1 activity during the course of the experiment are shown in Figure 3.

The measurements were performed as a single measurement, except on day 0 (duplicate measurements). For each measurement a new aliquot was taken. In the Figures, the PON1 activity and the HDL-C concentration have been expressed as percentage relative to the value obtained in fresh serum immediately after blood withdrawal (time point T = 0).

## Competing interests

None of the authors has any financial or non-financial competing interests.

## Authors’ contributions

PKB has made substantial contribution to the conception, acquisition of the data, analysis and interpretation of the data. MG has made important critical and intellectual contribution to the paper. EHJMJ has made a major contribution to the interpretation of the data and drafting and writing of the paper. All authors read and approved the final manuscript.
